# Exploring Teslasuit’s Potential in Detecting Sequential Slip-Induced Kinematic Changes among Healthy Young Adults

**DOI:** 10.3390/s23146258

**Published:** 2023-07-09

**Authors:** Jacob Hepp, Michael Shiraishi, Michelle Tran, Emmy Henson, Mira Ananthanarayanan, Rahul Soangra

**Affiliations:** 1 Crean College of Health and Behavioral Sciences, Chapman University, Orange, CA 92866, USA; jhepp@chapman.edu (J.H.); shiraishi@chapman.edu (M.S.); micheltran@chapman.edu (M.T.); ehenson@chapman.edu (E.H.); ananthanarayanan@chapman.edu (M.A.); 2 Fowler School of Engineering, Chapman University, Orange, CA 92866, USA

**Keywords:** perturbation training, fall risk, gait kinematics, wearable technologies

## Abstract

This study aimed to assess whether the Teslasuit, a wearable motion-sensing technology, could detect subtle changes in gait following slip perturbations comparable to an infrared motion capture system. A total of 12 participants wore Teslasuits equipped with inertial measurement units (IMUs) and reflective markers. The experiments were conducted using the Motek GRAIL system, which allowed for accurate timing of slip perturbations during heel strikes. The data from Teslasuit and camera systems were analyzed using statistical parameter mapping (SPM) to compare gait patterns from the two systems and before and after slip. We found significant changes in ankle angles and moments before and after slip perturbations. We also found that step width significantly increased after slip perturbations (*p* = 0.03) and total double support time significantly decreased after slip (*p* = 0.01). However, we found that initial double support time significantly increased after slip (*p* = 0.01). However, there were no significant differences observed between the Teslasuit and motion capture systems in terms of kinematic curves for ankle, knee, and hip movements. The Teslasuit showed promise as an alternative to camera-based motion capture systems for assessing ankle, knee, and hip kinematics during slips. However, some limitations were noted, including kinematics magnitude differences between the two systems. The findings of this study contribute to the understanding of gait adaptations due to sequential slips and potential use of Teslasuit for fall prevention strategies, such as perturbation training.

## 1. Introduction

Falls are the primary cause of injury among individuals aged 65 and above in the United States; approximately 27.5% of older adults reported falling in the previous year, and 10.2% reported an injury related to a fall [1]. Older adults who reported fall-related injuries experienced difficulty with various activities, including vision, cognition, walking or climbing stairs, performing errands alone, and dressing or bathing. Unfortunately, the incidence of falls among older ones increases with age, as 33.8% of individuals over the age of 85 reported falling in the past year, with 13.9% reporting an injury related to a fall [2]. As we age, several changes in gait may occur that can affect the risk of falling. Kerrigan et al. [3] identified several factors, such as decreased step length, increased cadence, and reduced terminal stance time (resulting in decreased peak hip extension and peak plantar flexion range of motion/power) [3]. Several gait parameters differ between fallers and non-fallers, including step length and cadence [4]. Fallers exhibit a significantly increased double limb stance time and variability that may contribute to increased fall risk [4]. Kwon et al. reported that fallers exhibit significantly reduced gait velocity, shorter step lengths, and longer stance times and that they require more time to reach peak vertical ground reaction force and mid-stance than non-fallers [5].

Even though weight-shifting errors and trips account for 62% of falls in long-term care facilities, slip recovery failures also commonly lead to falls [6]. A slip occurs when an individual’s center of mass (COM) is located outside their base of support, which can be compensated for by a corrective movement to avoid falling. Various gait abnormalities, including gait speed, stride time, step width/length, single and double limb stance times, and gait symmetry, may contribute to increased slip risk and fall incidents in individuals of all ages [7]. Mainly older adults with a stroke, gait asymmetry, increased stride time, reduced gait speed, and increased medial-lateral amplitude have an increased risk of falls [8,9]. For an intervention for slip-related falls, Pai et al. proposed enhancing dynamic stability and altering weight support can significantly increase the chances of a successful slip recovery through a compensatory backward step [10]. The same group discovered a strong correlation between increased gait speed and improved stability during a slip [11].

Numerous studies have developed predictive models using sensor data to accurately forecast falls and potentially identify optimal gait parameters to reduce the risk of falls [12]. Gait parameters such as step length, segment angles, the center of mass state, and ground reaction forces have been employed to construct a predictive model for slip-induced falls and reported a classification accuracy of 75.9% [13]. The investigators found that the right thigh angle at touchdown of the slipping foot, the maximum ground reaction force of the slipping limb after touchdown, and the momentum change from touchdown to recovery foot liftoff showed the most significant predictive power. Teslasuit is a full-body suit with embedded inertial sensors, haptics, and biometry technology. It features a motion capture system with 14 inertial measurement unit sensors for body tracking, allowing for comprehensive biomechanical analysis. Multiple studies have demonstrated the accuracy and efficacy of the Teslasuit in capturing spinal biomechanics and kinetics as well as in identifying and correcting movement errors. For example, Weber et al. reported that the Teslasuit accurately captured the spinal range of motion and speed in individuals with axial spondyloarthritis [14], while Caserman et al. demonstrated that it could detect and correct errors in real-time to improve functional movement during specific exercises [15]. In contrast, whether the Teslasuit is sensitive enough to capture slip-related subtle gait changes is still unclear. The current study aims to demonstrate that the Teslasuit can provide a more convenient and improved method for capturing slip-induced gait characteristics. The study will use perturbations to elicit slip-induced responses and capture and analyze gait characteristics using the Teslasuit.

## 2. Materials and Methods

This study aimed to evaluate whether the motion sensing abilities of the Teslasuit could detect slight variations in gait following slip perturbations similar to those seen by an infrared motion capture system. The Chapman University Institutional Review Board approved the study, and all participants signed written informed consent before participation. The inclusion criteria were subjects aged 18–35 years who could walk independently and did not have any prior injury at lower extremity.

Design of Repeated Slip Perturbations: The perturbations were delivered when subjects walked on computer-assisted rehabilitation environment (CAREN) systems, such as the Gait Real-time Analysis Laboratory (GRAIL). The virtual reality setup comprises a six-degrees-of-freedom motion platform (Moog Inc., East Aurora, NY, USA) and a 1.7 m long dual-belt-instrumented treadmill (Forcelink, B.V., Culemborg, The Netherlands) positi oned side-by-side, which is capable of high accelerations of up to 5 m/s^2^. The visual inputs were synchronized with the subject’s treadmill walking speed, creating the impression of walking on an endless pastoral path. To ensure uniformity, the preferred walking speed for each participant was obtained by incrementing the treadmill speed until the participant found it similar to their typical walking speed [16]. A total of 12 healthy adults (6 females and 6 males) participated in this study (refer to Table 1 for anthropometrics). Two Teslasuits—male and female, both of medium size—were used in this study. A Teslasuit consisting of 14 IMUs and a 34-reflective-marker set located on bony landmarks was worn by each participant. To avoid interference with normal walking and minimize the risk of injury in the event of a fall, the participants were fitted with a full-bodyweight-supported overhead harness during all walking and slip trials. The experiments were carried out using the Motek GRAIL, which allowed for the real-time detection of accurate timing of heel strikes during walking.

**Figure 1:**
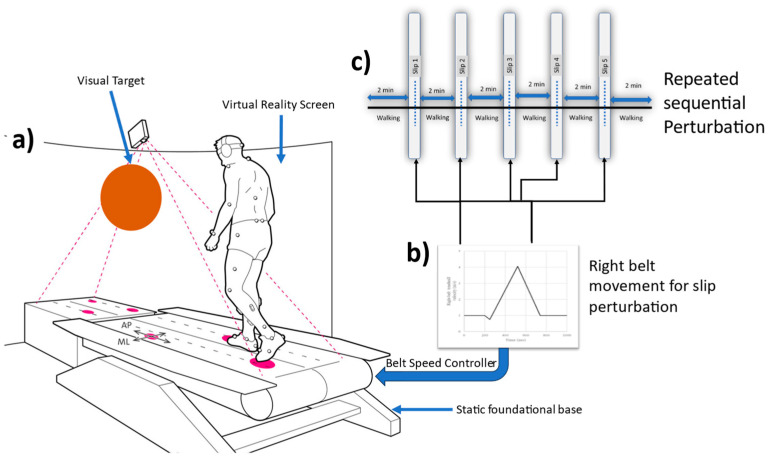
An overview of experimental protocol (**a**) participant walking on split-belt treadmill at constant normal preferred belt speed; (**b**) right belt speed was manipulated to induce slip five times during 12 min of walking. (**c**) Five repeated sequential slip perturbations were provided after 2 min of walking.

**Figure 2:**
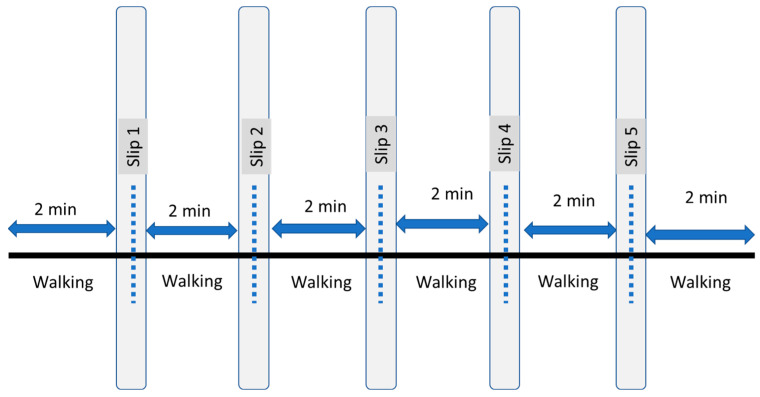
A 12 min walking protocol with five repeated perturbations occurring 2 min apart. The slips were induced during the heel strike event.

**Figure 3:**
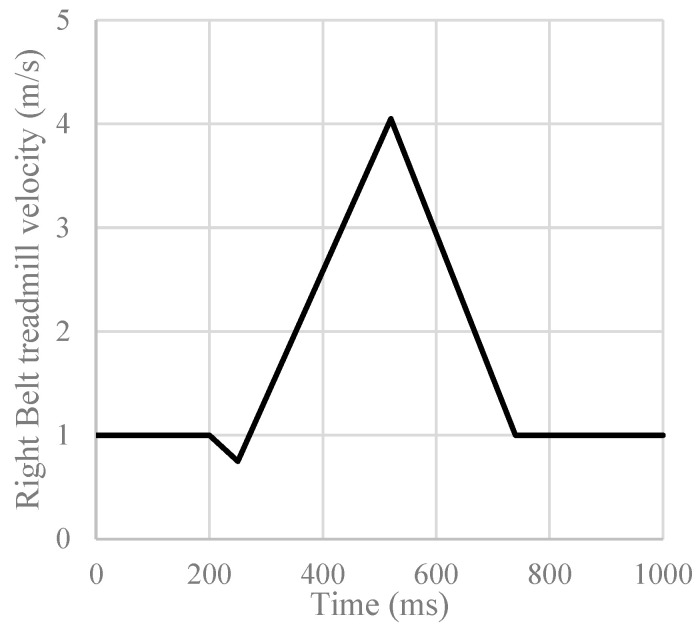
Graphical representation of the treadmill right belt speed to induce slip.

**Figure 4:**
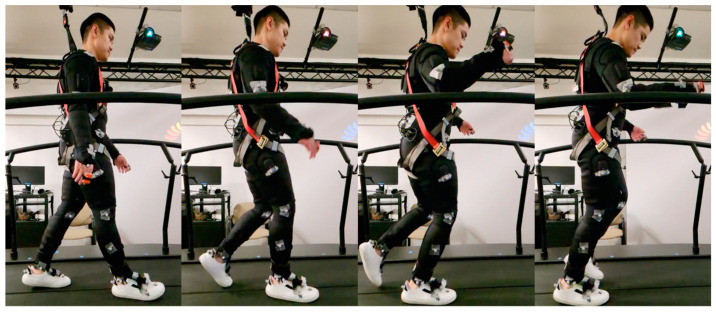
Participant walking during 12-min and experiencing slip perturbation trial condition.

All participants were given a visual target on a virtual reality screen and were asked to look at the target while walking. Each participant was instructed to perform a 5 min normal walking trial. All normal gait characteristics were evaluated from these trials, and they served as acclimatization before the perturbation walking trial. Subsequently, a 12 min walking trial was conducted, during which five intermittent slip perturbations were introduced, with approximately two-minute intervals between each perturbation. Figure 1 provides an overview of experimental protocol. The first perturbation was initiated at approximately 2 min, or 12,000 frames, after the start of the trial, followed by subsequent perturbations scheduled for 2 min, or 12,000 frames, after each perturbation (refer to Figure 2). This perturbation timing varied slightly on right heel contact timing. A protocol similar to that used by Sessoms et al. was adopted [17] (Figure 3). When a frame that was designated as a perturbation elapsed, the system would await a force on the right force plate that exceeded the preset threshold based on the participant’s weight. Once such a force was detected, the right treadmill belt would rapidly accelerate (15 m/s^2^) in the direction of motion, inducing a slip (refer to Figure 3 and Figure 4).

Data Processing: The motion capture data were analyzed using Vicon Nexus, while the Teslasuit Studio recorded and analyzed the data from Teslasuit. Gait analysis was conducted using the Gait Offline Analysis Tool (GOAT), and the gait pattern values were compared between the Teslasuit and motion capture systems. To differentiate kinematics before and after slip, statistical parameter mapping was used.

Statistical Parameter mapping (SPM): Statistical parameter mapping (SPM) is a widely used technique in neuroscience and medical imaging for analyzing brain activity and structure. It is a method for mapping statistical results from neuroimaging data onto a standardized anatomical template of the brain. SPM has several advantages for movement scientists and biomechanists. One of the primary advantages is that SPM does not require any abstraction of the original time series to perform statistical analysis. Instead, the entire 1D field can be examined using a non-directed hypothesis test without ad hoc assumptions regarding the spatiotemporal foci of interest. This is in contrast to other methods that require the visualization of the 1D time series and the extraction of a summary scalar for statistical analysis. SPM employs random field theory (RFT) to perform topological inference instead of separate inferential tests at each time point, which would result in an inflation of Type I error. RFT uses the local correlation between adjacent time points to mitigate the multiple testing problem and offers accurate sampling-rate independent control of Type I errors when testing correlated field data.

Rather than calculating a *p*-value at each time sample, SPM calculates a *p*-value for clusters of statistics that cross a critical threshold. SPM *p*-values are defined as the probability that smooth, random continua would produce a supra-threshold cluster as broad as the observed cluster. Critical thresholds are typically calculated with a Type I error of α = 0.05, and when the observed t-statistic time series crosses the threshold, this cluster has a *p* < 0.05, allowing the researcher to reject the null hypothesis H0 of no difference between the two time series. We used the open-source MATLAB package spm1d for this study.

## 3. Results

**Figure 5:**
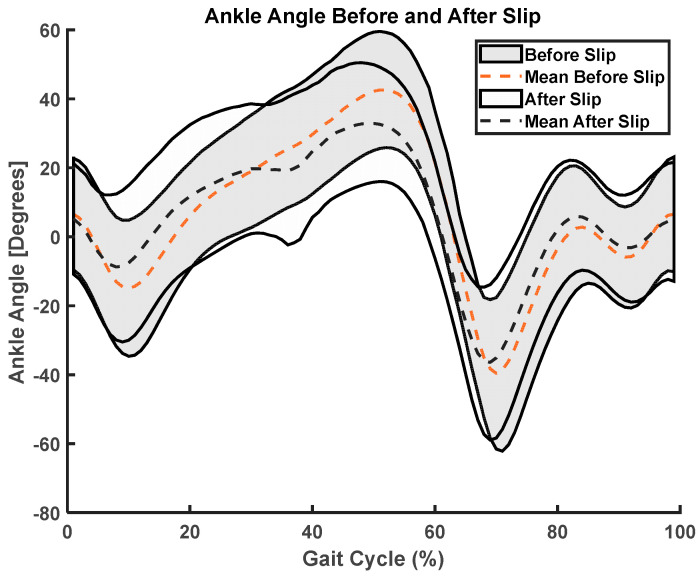
Before and after slip ankle angle; (i) mean (dotted) during a gait cycle, (ii) standard deviation (shaded).

**Figure 6:**
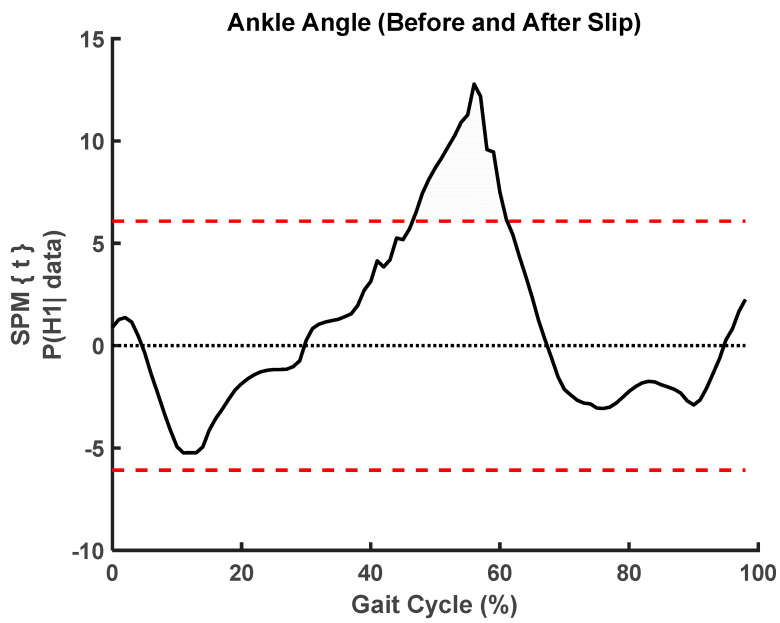
SPM analysis of ankle angle curves before and after slip. The shaded region shows significant differences from 45% of gait cycle to 65% of gait cycle.

**Figure 7:**
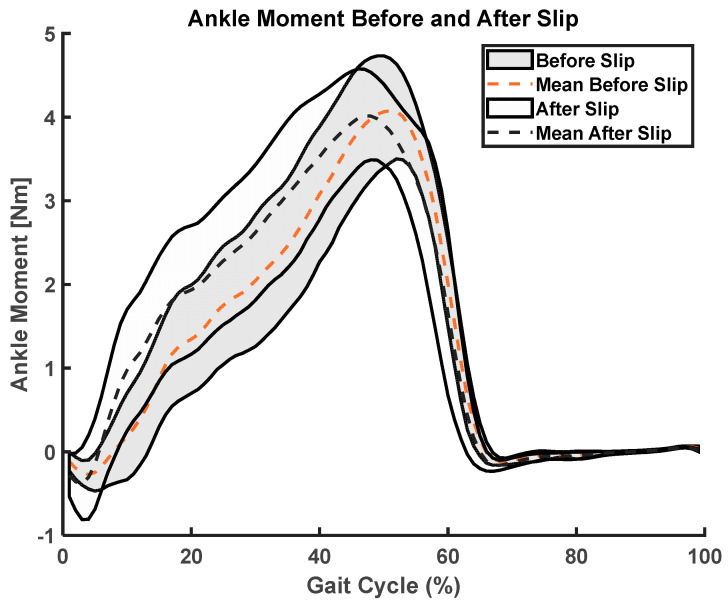
Before and after slip ankle moments (i) mean ankle moments represented as dotted lines during a gait cycle, (ii) standard deviation shown as shaded regions bounding the mean.

**Figure 8:**
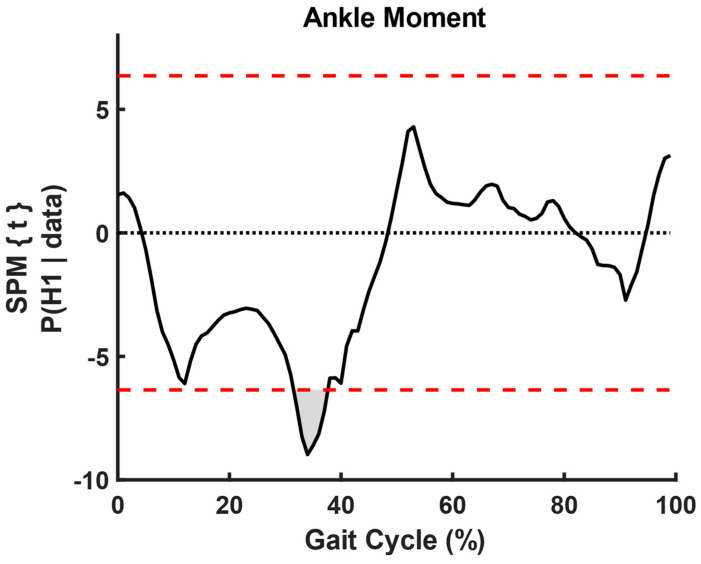
SPM analysis of ankle moment curves before and after slip. The shaded region shows significant differences from 30% of gait cycle to 40% of gait cycle. The dotted red lines show 95% confidence upper and lower bounds. The black dotted line shows zero SPM between the curves.

We used paired *t*-test SPM analysis and found significant changes in ankle angle (t = 6.08, *p* < 0.001, fwhm = 7.58) and moment (t = 6.35, *p* < 0.001, fwhm = 6.31) before and after slip, where fwhm stands for full width at half maximum of the Gaussian kernel (Figure 5, Figure 6, Figure 7 and Figure 8).

**Figure 9:**
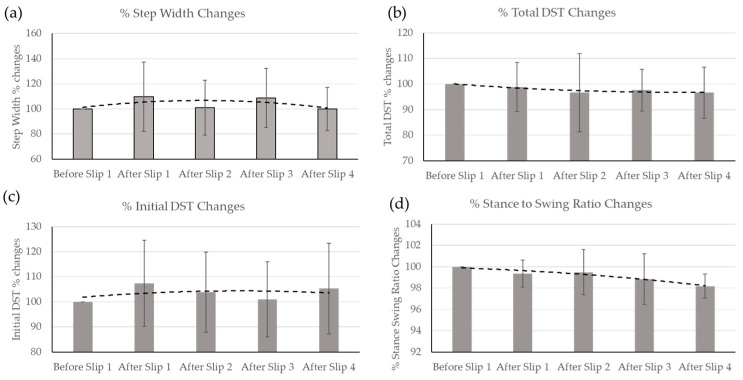
Percent change in gait parameters from initial (before slip 1 perturbation) values were evaluated for (**a**) step width, (**b**) total DST, (**c**) initial DST, (**d**) stance to swing ratio. The dotted lines show trend followed after sequential perturbations.

Considering gait parameter initial values at 100% before the first slip, the percent change in step width, double support time, initial double support time (a component of double support time), and stance-to-swing ratio times were evaluated and shown in Figure 9 below.

Our study employed statistical parameter mapping to compare the kinematic curves of ankle, knee, and hip movements obtained from the Teslasuit and a camera-based motion capture system. Surprisingly, our analysis did not reveal statistically significant differences between the two systems.

We performed a matched paired *t*-test to compare the measures of initial double support, step width, total double support, and stance-to-swing ratio for before and after slip events. Our analysis revealed significant differences in these variables. First, we observed a significant difference in initial double support (t(12) = 2.96, *p* < 0.01), indicating a change in the duration of the initial double support phase following slip events. Second, we found a significant difference in step width (t(12) = −2.03, *p* = 0.03), suggesting a change in the width of steps taken after slip events. Lastly, we identified a significant difference in total double support (t(12) = 2.31, *p* = 0.03), indicating a change in the total duration of the double support phase before and after slip events (Table 2). We did not find significant differences in stance-to-swing ratio. These findings demonstrate that slip events significantly impact gait parameters related to initial double support, step width, and total double support. The results suggest that individuals modify their gait pattern following slips to enhance stability and reduce the risk of subsequent falls. It is important to note that the reported *p*-values are based on a two-tailed test, and the degrees of freedom (df) are indicated in parentheses. The significance level (α) was set at 0.05.

**Figure 10:**
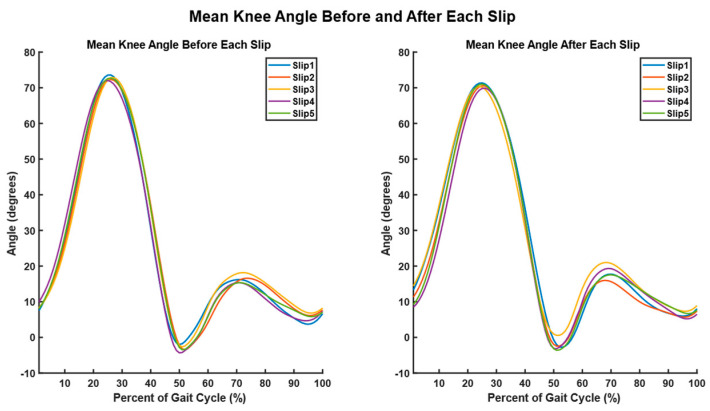
Representative graphs showing knee angles before slip and knee angles after slip from one participant. The different colors represent different slip conditions.

**Figure 11:**
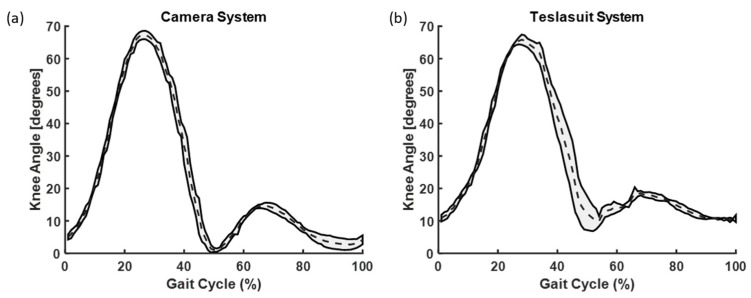
A comparative graph of knee angles evaluated from (**a**) camera system and (**b**) Teslasuit. The dotted lines represent the mean and shaded area represent the standard deviations at that time.

**Figure 12:**
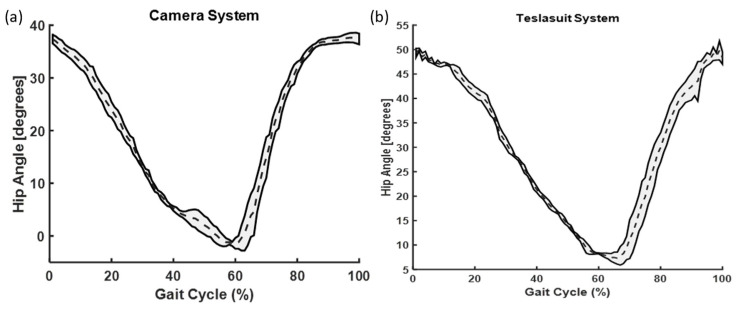
A comparative graph of hip angles evaluated from (**a**) camera system and (**b**) Teslasuit. The dotted lines represent the mean and shaded area represent the standard deviations at that time.

We conducted SPM analysis for knee (Figure 10, Figure 11 and Figure 12) and hip angles before and after each slip condition, but no significant differences were found. In comparing the knee angles of the Teslasuit and camera system, we found that the Teslasuit reported slightly higher knee (Figure 11) and hip flexion angles (Figure 12) than the camera system, but they were not found to be significantly different for both systems using SPM.

## 4. Discussion

The emergence of wearable technologies has opened up new possibilities for enhancing training curricula and developing more personalized treatment and rehabilitation programs. With new advancements in wearable technologies, quick measurement and analysis of biomechanical data are feasible. One of the most promising technologies is the Teslasuit, which incorporates haptic feedback, electro muscle stimulation, and transcutaneous electrical nerve stimulation; can capture and analyze body movements; and can probably intervene in real time during a perturbation event. The suit is used in high-risk work training, physical training, and stroke rehabilitation [15,18,19,20]. Several recent studies have investigated the efficacy of perturbation training in reducing fall risk in older adults. In particular, Bhatt et al. found that consecutive trip perturbation training led to higher toe clearance with improved trip adaptation [11] and reduced fall risk. The Teslasuit’s real-time motion analysis capabilities make it a promising tool for measuring fall risk and training for fall recovery. Gait analysis can be performed without needing location-locked motion capture cameras, allowing for a more practical and efficient method of identifying gait problems and areas for improvement. This is especially useful when considering repeated perturbation training, which improves resistance to future falls through improved stability and limb support control and retention of these skills through motor memory. Fall resistance is expected to improve after a single exposure to perturbation, reducing fall incidence to 0% and improving gait stability in older adults without altering gait speed [21,22]. Identifying issues in the recovery process can help improve overall fall susceptibility, particularly with task-specific training [23]. The Teslasuit’s ability to gather kinematics data could prove valuable when evaluating fall risk and preventing falls in older adults. If the Teslasuit’s movement assessment capabilities prove to be consistent with established analysis methods, such as infrared motion capture systems, the suit could become a valuable resource in diagnosing and treating various illnesses and injuries [19]. It may also have potential applications in hazardous locations associated with military and space efforts due to its sensor capabilities and ability to detect slips and falls. While it is unclear whether the Teslasuit is sensitive enough to capture slip characteristics, previous research by Weber et al. and Caserman provides evidence of the reliability and validity of its measurement capabilities [14,15].

This study utilized statistical parameter mapping to evaluate and compare the kinematic profiles of ankle, knee, and hip movements captured by the Teslasuit and a camera-based motion capture system. Interestingly, our analysis did not uncover any noteworthy statistical differences between the kinematics of the two systems. This suggests that the Teslasuit may offer a viable alternative to the camera-based motion capture system in out-of-laboratory environments for assessing ankle, knee, and hip kinematics during slips and could intervene timely. However, it is essential to consider some limitations. Firstly, the absence of significant differences does not necessarily indicate that the two systems are equivalent in all aspects. The systems may differ in other factors, such as accuracy, precision, or specific movement conditions. Secondly, the absence of significance could also be influenced by sample size, variability within the data, or other methodological factors. We found that the Teslasuit reported slightly higher knee flexion angles compared to camera-based systems (Figure 10), but these values were not found to be significantly higher. We also compared kinematics before and after slip perturbations, and SPM analysis revealed ankle angles and moments significantly changed after slip perturbation (Figure 4, Figure 5, Figure 6 and Figure 7).

We found that step width increases immediately after the slip and represents reactive compensatory adaptations the participants made to regain their balance by increasing their base of support. There was a sustained increase in step width when the participant continued to walk (as shown in Figure 9a). Still, this increase slowly began to decrease, eventually beyond the initial step widths recorded before the first slip occurred. This may be due to adaptations and learning effects of the slip. Since the slip itself does not change throughout the trial, each participant was able to become accustomed to it, learn, and recover faster, decreasing their need to keep their base of support larger and a greater reliance on visual or vestibular information to remain balanced.

We also found that total double support times decreased immediately following perturbation and throughout the trial (Figure 9b). In recovery from the perturbation, the initial double support time increased (Figure 9c) as participants stumble (during perturbation) and have to keep pace with the treadmill’s set speed. This double support time remains lowered, as participants rely on visual information rather than somatosensory information to recover from slips. A similar trend to that of total double support time was seen in the stance-to-swing ratio (Table 2).

Our results imply that the Teslasuit may be a reliable alternative to the camera-based system for capturing and analyzing kinematic data related to perturbation-related kinematic changes at ankle, knee, and hip joints. The Teslasuit can be used for slip detection. It can quickly detect a change in gait parameters representing a fall risk and promptly intervene in these scenarios. The suit’s ability to detect these changes enables it to be of value in various rehabilitation settings, such as stroke rehabilitation programs and fall intervention programs involving repeated perturbation training.

## 5. Conclusions

Wearable technologies like the Teslasuit offer new opportunities for personalized treatment and rehabilitation. The Teslasuit’s real-time motion analysis capabilities make it valuable for measuring fall risk and training for fall recovery. Our study compared Teslasuit and camera-based motion capture system kinematic data and found no significant differences, suggesting the Teslasuit’s viability in out-of-laboratory environments. However, limitations and potential differences in accuracy and precision should be considered. Perturbation-induced kinematic changes were accurately captured by the Teslasuit, including adaptations in step width and double support times. The Teslasuit offers a reliable and practical solution for capturing and analyzing kinematic data related to perturbation-induced changes in ankle, knee, and hip joints in and outside lab environments. Further research is needed to explore its full potential and address the remaining limitations.

## Figures and Tables

**Table 1:** Anthropometric data for participants (mean± standard deviation).

Sex	Age (Years)	Height (cm)	Weight (kg)	BMI (kg/m^2^)
Female (*n* = 6)	26.2 ± 0.9	153.4 ± 4.6	56.2 ± 8.8	23.7 ± 2.7
Male (*n* = 6)	26.6 ± 1.3	171.6 ± 4.9	72.6 ± 10.7	24.5 ± 2.8

**Table 2:** Gait parameter changes before and after perturbation (slip). Where * represents significant differences.

Gait Parameters	Before-Slip	After-Slip	*p*-Value
Cadence	103.8 ± 9.45	110.2 ± 17.11	0.20
Initial Double Support % *	17.05 ± 0.88	15.57 ± 0.98	0.01 *
Single Support Time %	33.16 ± 1.456	34.61 ± 1.837	0.06
Stance to Swing % Ratio	67.28 ± 1.067	66.34 ± 0.969	0.05
Stance Time (s)	0.791 ± 0.075	0.749 ± 0.067	0.14
Step Length (m)	0.510 ± 0.216	0.491 ± 0.211	0.43
Step Time (s)	0.586 ± 0.053	0.562 ± 0.063	0.23
Step Width (m) *	0.141 ± 0.018	0.161 ± 0.019	0.03 *
Stride Length (m)	1.013 ± 0.446	0.990 ± 0.439	0.46
Stride Time (s)	1.175 ± 0.099	1.128 ± 0.100	0.19
Swing Time (s)	0.384 ± 0.026	0.379 ± 0.035	0.38
Terminal Double Support %	17.07 ± 1.533	16.17 ± 1.172	0.11
Total Double Support % *	34.12 ± 2.115	31.74 ± 1.715	0.01 *

## Data Availability

The raw data supporting the conclusions of this article will be made available by the corresponding author upon reasonable request.
